# Current understanding of metal-dependent amyloid-β aggregation and toxicity

**DOI:** 10.1039/d2cb00208f

**Published:** 2022-11-22

**Authors:** Yelim Yi, Mi Hee Lim

**Affiliations:** a Department of Chemistry, Korea Advanced Institute of Science and Technology (KAIST) Daejeon 34141 Republic of Korea miheelim@kaist.ac.kr

## Abstract

The discovery of effective therapeutics targeting amyloid-β (Aβ) aggregates for Alzheimer's disease (AD) has been very challenging, which suggests its complicated etiology associated with multiple pathogenic elements. In AD-affected brains, highly concentrated metals, such as copper and zinc, are found in senile plaques mainly composed of Aβ aggregates. These metal ions are coordinated to Aβ and affect its aggregation and toxicity profiles. In this review, we illustrate the current view on molecular insights into the assembly of Aβ peptides in the absence and presence of metal ions as well as the effect of metal ions on their toxicity.

## Abstract 2

This review describes the effects of metal ions on the aggregation and toxicity profiles of Aβ.

## Graphical abstract

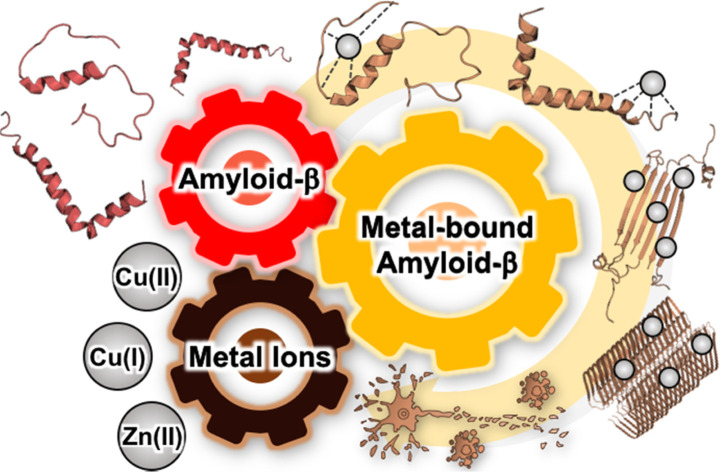

## Introduction

Globally, over 55 million people are living with dementia associated with population aging.^1,2^ Alzheimer's disease (AD) is the most common form of dementia that is neuropathologically characterized by the accumulation of protein aggregates, such as amyloid-β (Aβ) aggregates.^3^ The amyloid cascade hypothesis claims Aβ as a primary causative factor of AD and, thus, research efforts have been made towards developing therapeutic agents that can control Aβ species.^4,5^ The development of human monoclonal antibodies that target Aβ aggregates further supports this hypothesis. For example, aducanumab was approved as the first disease-modifying treatment for AD by the United States Food and Drug Administration.^5,6^ Unfortunately, the use of aducanumab against AD is questionable. Very recently, lecanemab was tested in the phase III clinical trial, demonstrating that it has better safety and efficacy than aducanumab.^7–9^ In addition, there has been a call for the expansion or modification of the amyloid cascade hypothesis based on emerging evidence on the inter-relationship between Aβ and other pathogenic elements found in AD-affected brains.^3,10,11^

Given that high concentrations of metals (*e.g.*, 0.4 mM for copper and 1.0 mM for zinc) are observed in senile plaques mainly composed of Aβ aggregates, metal ions and Aβ are suggested to be mutually involved in the pathogenesis of AD.^3,12–17^ Labile metal ions, such as Cu(i/ii) and Zn(ii), can be released into the synaptic cleft and bind to Aβ peptides forming metal–Aβ complexes.^18–20^ Such complexation between metal ions and Aβ can affect its aggregation pathways producing toxic Aβ aggregates (*e.g.*, soluble and structured oligomers).^21–26^ Furthermore, redox-active metal ions [*e.g.*, Cu(i/ii)] bound and unbound to Aβ can catalytically generate reactive oxygen species (ROS) and induce oxidative stress leading to neuronal cell death. Based on these reactivities, numerous studies have attempted to elucidate the Aβ-related pathology associated with metal ions at the molecular level.^3,10–12,18^ In this review, we illustrate the aggregation pathways of Aβ in the absence and presence of metal ions, with an emphasis on the molecular mechanisms underlying its self-assembly. The influence of metal ions on the toxicity profile of Aβ is also described. Overall, this review provides insight into the roles of metal ions in the pathogenic characteristics of Aβ based on bioinorganic chemistry.

## Aggregation of Aβ peptides

**Fig. 1:**
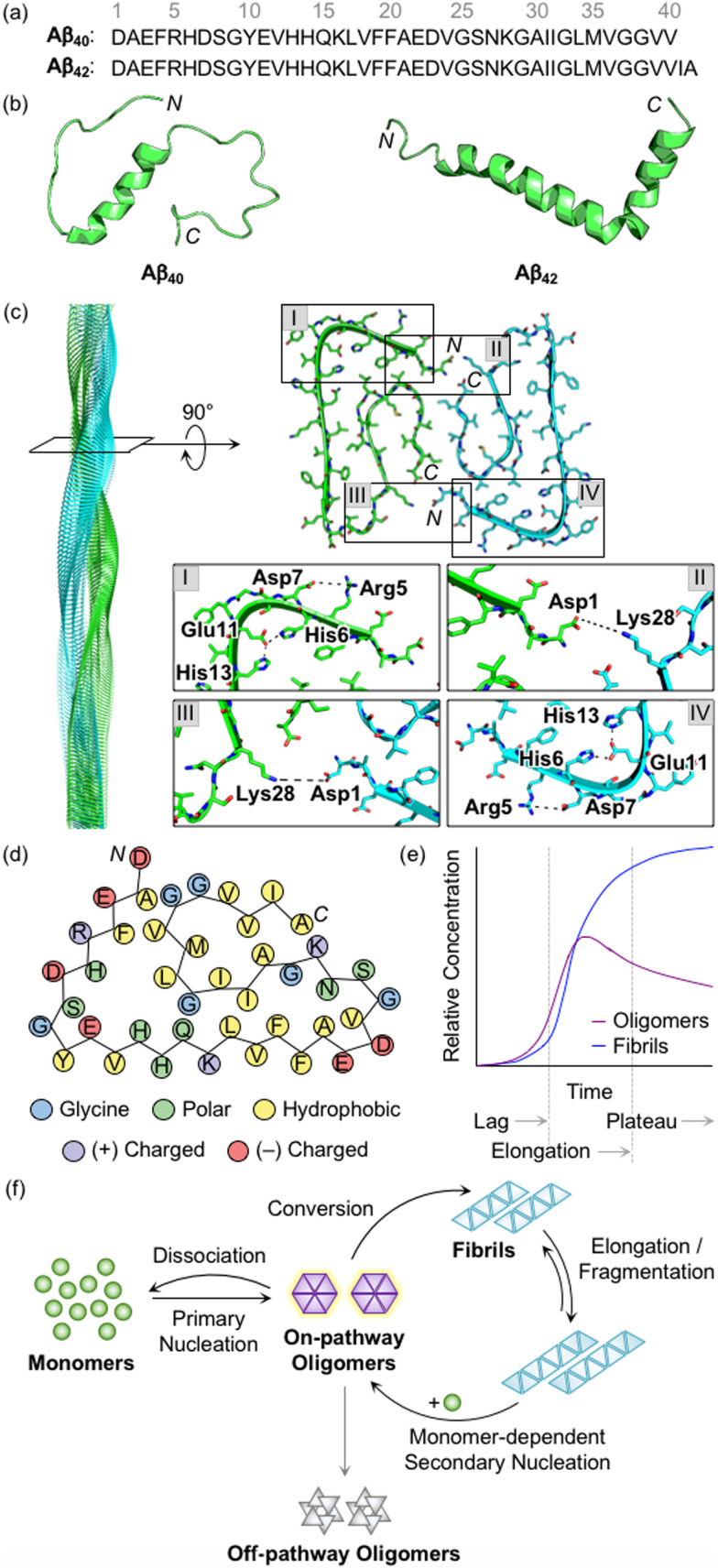
Structures of Aβ peptides and their aggregation. (a) Amino acid sequences of Aβ_40_ and Aβ_42_. (b) Examples of previously reported Aβ monomers (for Aβ_40_, PDB 2LFM;^30^ for Aβ_42_, PDB 1IYT^31^). (c) Example of a previously reported Aβ_42_ fibril (PDB 1IYT^31^). The salt bridges that contribute to the stabilization of fibrillar forms are indicated in I–IV. (d) Schematic view of a fibrillar structure of Aβ_42_ with amino acid residues colored according to their polarity and charge states. Three His residues in Aβ are classified into polar amino acid residues based on its chemical environment at pH 7.4. (e) Schematic representation of relative concentrations of oligomeric and fibrillary Aβ as a function of time.^52^ The increase in the concentration of fibrils in a sigmoidal manner presents the macroscopic aggregation of Aβ that is typically divided into lag, elongation, and plateau phases. Reproduced with permission from ref. 52. Copyright© 2020 Springer Nature. (f) Schematic illustration of microscopic steps involved in Aβ aggregation.^37^ Reproduced with permission from ref. 37. Copyright© 2022 AIP Publishing.

Aβ peptides with 38–43 amino acid residues in length are generated by the proteolysis of amyloid precursor protein (APP).^3,27,28^ β- and γ-Secretases cleave APP at extracellular and intracellular regions, respectively, mainly producing two Aβ isoforms, Aβ_40_ and Aβ_42_ (Fig. 1a).^29^ Aβ peptides are intrinsically disordered and, thus, their three-dimensional structures have not been fully determined.^3,29^ A large amount of research has been dedicated to finding optimal experimental conditions that can stabilize Aβ monomers. For example, Ramamoorthy's group reported a structure of monomeric Aβ_40_ in aqueous media that was identified by high-resolution solution nuclear magnetic resonance (NMR) spectroscopy.^30^ As presented in Fig. 1b, the central region (*i.e.*, His13−Asp23) of Aβ was revealed to form a 3_10_ helix. The *N*- and *C*-terminal regions relatively lacked structured conformations, but they were not completely unstructured, as local interactions between the side chains of amino acid residues beside the helical region generated twists and turns. In addition, the structure of monomeric Aβ_42_ was characterized in the cellular membrane-mimicking apolar environment by solution NMR and circular dichroism (CD) spectroscopies.^31^ In a mixture of water and hexafluoroisopropanol, the Aβ_42_ monomer showed two helical regions at Ser8−Gly25 and Lys28−Gly38 that are connected by a β-turn. Aβ monomers aggregated into amyloid fibrils with a conformational transition of helical regions into β-strand structures.^3,28,29^ Each β-strand was perpendicularly organized to a fibril axis, as shown in Fig. 1c. Multiple driving forces, including hydrophobic contacts between hydrophobic amino acid residues in the central and *C*-terminal regions, intermolecular hydrogen bonds, and salt bridges, can contribute to forming and stabilizing Aβ fibrils, as illustrated in Fig. 1c and d.^28,29,32,33^

During the aggregation processes, heterogeneous Aβ aggregates are generated with variable sizes and morphologies.^3,12,28^ As indicated in Fig. 1e, profiling the aggregation kinetics of Aβ at the macroscopic level portrays three stages: lag, elongation, and plateau phases.^3^ The overall aggregation pathways of Aβ can be further analyzed at the microscopic level, as depicted in Fig. 1f.^34–39^ The assembly of monomers is initiated by the primary nucleation step that generally presents the step for forming nuclei from monomers. Nuclei are often delineated as the smallest aggregates for which the addition of monomers preferably occurs rather than the loss of monomers.^40,41^ The definition of oligomers, which possibly correspond to nuclei, is different depending on the literature. The aggregates, except for monomers and fibrils, are broadly regarded as oligomers.^19^ In particular, oligomers may refer to aggregates with certain sizes (*e.g.*, from dimer to triacontamer), aggregation rates, or morphologies, distinct from those of fibrils.^40,42^ In some reports, oligomers can be specific depending on experimental systems, such as preparation procedures and analytic methods.^40,43,44^ It has not been clear which oligomeric species could be nuclei. Following the aggregation, oligomers can be converted into fibrils that are β-sheet-rich aggregates.^37^ Through the elongation and fragmentation steps (Fig. 1f), the size of fibrils is varied by providing new ends of the length extension and breaking down the fibrils, respectively.^34–38^ These fibrils can be offered as surfaces that catalyze the generation of new nuclei (*e.g.*, oligomers) *via* secondary nucleation.^45–47^ The aggregation of Aβ could include a process of monomer-dependent secondary nucleation, whereby monomers preferentially generate nuclei on the surface of preformed aggregates.^35,36,48^

In the case of oligomers as intermediates for Aβ aggregation, they can be subdivided into two classes: on-pathway or off-pathway oligomers (Fig. 1f).^26,49,50^ When oligomers aggregate into fibrils, the process can be regarded to follow the on-pathway aggregation. On the other hand, oligomers generated through alternative aggregation pathways are accepted to be classified as off-pathway oligomers. It should be noted that this binary definition for oligomers could be unclear because oligomers in a heterogeneous population undergo multiple fates that cannot be definitely specified.^51^ For example, some oligomers can be dissociated back to monomers, categorized as neither on-pathway oligomers nor off-pathway oligomers. The competition between the dissociation of oligomers and the formation of fibrils was supported by the studies of aggregation kinetics that measure the concentrations of oligomers and fibrils, as represented in Fig. 1e.^52,53^ The results obtained by the mathematical fitting of rate equations to aggregation curves denote that less than 10% of oligomers are converted into fibrillar species, whereas the others disassemble into the monomers. Knowles and coworkers showed a non-binary and quantitative definition in which an oligomer is assigned with a value (*e.g.*, between 0 and 1) that describes its relative contribution to fibril formation, rather than the binary definition for oligomers, which may be more appropriate to establish a general concept for the aggregation of Aβ.^51^ Moreover, research progress has been made towards connecting the results from macroscopic and microscopic analyses for the mechanisms of Aβ aggregation, which suggests that multiple microscopic steps can take part in one macroscopic aggregation phase.^40^ It should be noted that in addition to the heterogeneous nature of intrinsically unstructured Aβ, the analysis of its self-assembly is challenging because of its different aggregation behaviors depending on experimental conditions (*e.g.*, the purity and concentration of Aβ, pH, ionic strength, and temperature).^41^

## Change in the properties of Aβ through interactions with metal ions

### Metal coordination to Aβ

**Fig. 2:**
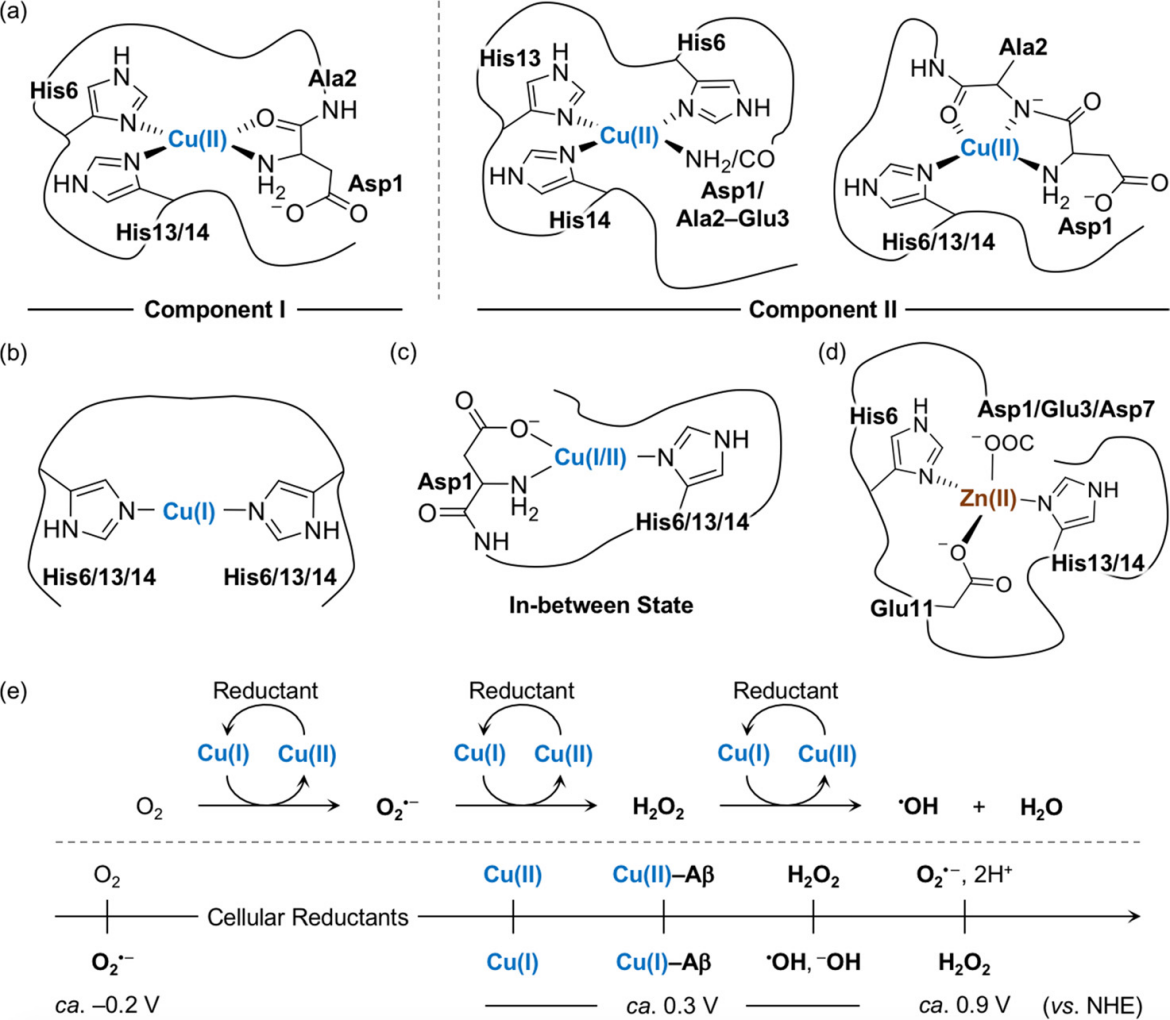
Metal-binding properties of Aβ. Examples of structures of (a) Cu(ii)–Aβ, (b) Cu(i)–Aβ, (c) their in-between state, and (d) Zn(ii)–Aβ. Possible fifth ligands on the metal centers are omitted in the figure for clarity. (e) Scheme of ROS formation catalyzed by Cu(i/ii) in the presence of a reductant and redox potentials of the species involved in the reactions with respect to the normal hydrogen electrode (NHE).^71^ Cellular reductants include ascorbate and glutathione.

The amino acid residues in Aβ responsible for metal binding have been identified by multiple biophysical methods (Fig. 2a–d). In the case of Cu(ii) with the d^9^ electronic configuration, two components of Cu(ii)–Aβ complexes were determined at different pHs by electron paramagnetic resonance (EPR), CD, electronic absorption, X-ray absorption, and NMR spectroscopies.^19,54,55^ At physiological pH, the *N*-terminal primary amine, two imidazole nitrogen (N) donor atoms from His6 and His13 or His14, and the oxygen (O) donor atom from the backbone carbonyl group between Asp1 and Ala2 coordinate to the Cu(ii) center, as shown in component I (3N1O coordination; Fig. 2a).^54,56–60^ Cu(ii)–Aβ complexes have a distorted square planar or square pyramidal geometry possibly with a weakly bound carboxylate group from the side chain of Asp1, Glu3, Asp7, or Glu11 or a water molecule at the apical position as a fifth ligand.^54,56–58,61,62^ The dissociation constant (*K*_d_) value of the Cu(ii)–Aβ complex was reported in a nanomolar range.^63–65^ The second Cu(ii) binding to Aβ could occur with at least two orders of magnitude weaker binding affinity, relative to the first Cu(ii) coordination; however, the second Cu(ii)-binding site has not been established.^66–69^ At relatively high pH (*ca.* pH ≥ 8), several potential Cu(ii)-binding sites in component II were suggested that include a deprotonated backbone amide moiety between Asp1 and Ala2: (i) 4N coordination with His6, His13, and His14 and either the *N*-terminal primary amine or the deprotonated backbone amide group between Asp1 and Ala2;^67,70^ (ii) 3N1O coordination with three N donor atoms (imidazole N donor atoms from His6, His13, and His14 or the *N*-terminal primary amine, the deprotonated backbone amide group between Asp1 and Ala2, and one imidazole N donor atom from His6, His13, or His14) and one O donor atom from the backbone carbonyl moiety between Ala2 and Glu3.^54,56–58,60^ The carboxylate group from the side chain of Asp1, Glu3, Asp7, or Glu11 is proposed to be at the apical position as the fifth ligand.^54,56,57,71^ This variation in the coordination sphere of Cu(ii)–Aβ complexes depending on the pH can be a factor for altering Aβ aggregation (*vide infra*).

Cu(i) coordination to Aβ may occur *via* three different combinations of His6, His13, and His14 in a linear geometry, as illustrated in Fig. 2b.^55,59,72–74^ Since there are distinct geometries between Cu(ii)–Aβ (*i.e.*, distorted square planar geometry) and Cu(i)–Aβ (*i.e.*, linear geometry), a large reorganization energy (*λ* = *ca.* 1.4 eV) is required for efficient electron transfer.^75^ To overcome this energy demand, an intermediate species (Fig. 2c) that is in equilibrium between Cu(ii)–Aβ and Cu(i)–Aβ has been proposed as a redox-competent state in which binding modes of Cu(i) and Cu(ii) are similar and, consequently, the reorganization energy for one-electron transfer (*λ* = 0.3 eV) is much less. In the cellular environment that includes reducing agents (*e.g.*, ascorbate and glutathione), Cu(ii) with and without Aβ can be reduced to Cu(i), as shown in Fig. 2e.^59,73,76,77^ Cu(i)–Aβ can react with O_2_ and, subsequently, produce ROS (*e.g.*, O_2_^⋅–^, H_2_O_2_, and ⋅OH) that can oxidatively modify Aβ with a consequent change in its aggregation.^71,78–81^ A wide range of *K*_d_ values for Cu(i)–Aβ (femtomolar to submicromolar) was reported depending on experimental conditions.^82,83^

Zn(ii) is a d^10^ metal ion that exhibits less structural variations upon complexation with Aβ, compared to Cu(ii). Possible binding modes of Zn(ii)–Aβ complexes have been determined by NMR and X-ray absorption spectroscopies.^55,84,85^ As displayed in Fig. 2d, Zn(ii) binding to Aβ at pH 7.4 can form a tetrahedral geometry through two imidazole N donor atoms from His6 and His13 or His14 and two O donor atoms from the carboxylate groups in the side chains of Asp1, Glu3, or Asp7 and Glu11.^84,85^ A water molecule is able to replace Asp1, Glu3, or Asp7.^84^ The *K*_d_ value of Zn(ii)–Aβ was reported to be in a micromolar range.^65,86^

It should be noted that the amino acid residues participating in metal coordination can be varied depending on the aggregation states of Aβ. Very limited studies indicated that the ligands that do not participate in metal coordination in monomers, such as carboxylate groups from the side chain of Glu3, Glu11, Glu22, or Asp23 and the *C*-terminus, are possibly involved in metal binding to Aβ aggregates.^87,88^ In the case of Aβ aggregates, metal ions may have distinct binding modes, compared to those found in Aβ monomers, since the alignment of peptides in the aggregates can induce intermolecular interactions, which may form metal-binding sites between peptides.^88–93^ The structural analysis of metal-bound Aβ aggregates would be further carried out in detail to advance our understanding of metal-associated Aβ aggregation.

### Metal-to-peptide stoichiometry

**Fig. 3:**
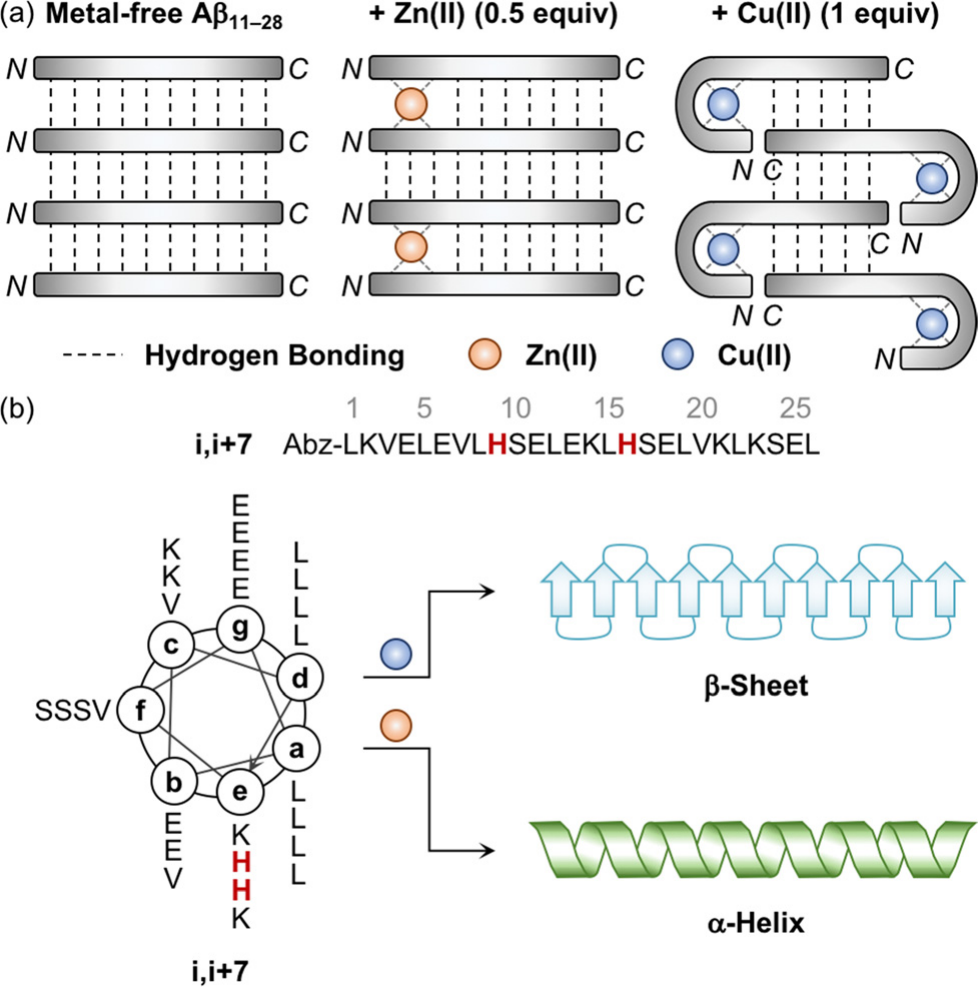
Influence of metal ions on the alignment and conformation of model peptides. (a) Possible metal-binding modes of Aβ_11–28_ depending on the type of metal ions and the metal-to-peptide stoichiometry.^95^ The sub-equimolar concentration of Zn(ii) can bridge two Aβ_11–28_ peptides, stabilizing their interaction and preferably forming β-sheets. In the presence of an equimolar amount of Cu(ii), Aβ_11–28_ can wrap around the metal ion and, consequently, reduce the number of hydrogen bonds, which possibly hinders the alignment required to form β-sheets. Reproduced with permission from ref. 95. Copyright© 2010 Springer Nature. (b) Conformational change of the coiled coil peptide (*i.e.*, i,i+7) upon the addition of metal ions. A helical wheel diagram presents the amino acid residues of the coiled coil peptide in a heptad repeat labeled as (a–g). The His residues that participate in metal binding are highlighted in red. Abz introduced at the *N*-terminal of the peptide indicates *o*-aminobenzoic acid. Cu(ii) contributes to the conversion of the coiled coil peptide into the β-sheet-rich peptide, while Zn(ii) stabilizes the α-helical structure of the peptide.

The aggregation of Aβ is shown to be significantly varied depending on the metal-to-Aβ ratio. For example, the sub-equimolar metal ion possibly bridges two peptides, which triggers the dimerization of Aβ.^90^ These dimers can serve as a nucleus that facilitates the aggregation of Aβ. This concept was experimentally demonstrated employing a model peptide, Aβ_11–28_, that contains a hydrophobic region (*i.e.*, Leu17–Ala21) responsible for initiating the aggregation by hydrophobic interactions and metal-binding sites.^94–96^ As presented in Fig. 3a, when sub-stoichiometric Zn(ii) was treated with Aβ_11–28_, the formation of the Aβ_11–28_ dimer bridged by Zn(ii) through His13 and His14 was observed by X-ray absorption and NMR spectroscopies.^95,96^ Upon incubation, amyloid fibrils were produced, confirmed by transmission electron microscopy (TEM) as well as the turbidity and fluorescence assays. This suggests that the fast exchange of Zn(ii) between monomers and aggregates enables the generation of nuclei, accelerating the formation of amyloid fibrils. In the presence of an equimolar concentration of metal ions, two metal–Aβ complexes could be conceptually aligned by hydrogen bonds between the backbone amide groups, which could eventually form β-sheets. When 1 equivalent of Cu(ii) was bound to the NH_2_-Xxx-Xxx-His motif at the *N*-terminal region of Aβ_11–28_, however, the number of hydrogen bonds decreased and, consequently, lowered the fibril formation, as monitored by the turbidity and fluorescence assays and TEM.^94^ When the metal-to-Aβ stoichiometry was above 1, an excess amount of metal ions can nonspecifically bind to Aβ peptides, which could deform hydrogen-bond networks between backbone amide groups and, thus, inhibit the production of amyloid fibrils, resulting in amorphous aggregates.^90^ It should be noted that pathophysiologically relevant metal-to-peptide stoichiometry was not clearly defined because of the difficulty in determining exact concentrations of heterogeneous Aβ species and metal ions that are dependent on the diseased state.^13,94,97,98^ Further investigations would be valuable to elucidate the metal-binding properties of various-sized Aβ peptides with different metal-to-peptide ratios.

### Charge state of Aβ

Given that hydrophobic interactions between Aβ peptides are the main driving force towards their self-assembly, the peptides with an overall charge close to 0 would be more favorable for their rapid aggregation.^12,19,90^ At physiological pH, the overall charge state of Aβ is 3– (Fig. 1d) and, thus, Cu(i/ii) or Zn(ii) binding to Aβ results in a net charge state nearer to 0, which can make metal–Aβ complexes more prone to aggregation. In addition, metal binding to Aβ lowers the p*K*_a_ values (*K*_a_, acidity constant) of the ligands from the peptide and, consequently, deprotonates the amino acid residues (*e.g.*, backbone amide group), decreasing the net charge, as depicted in component II (Fig. 2a).^67,70^ Zn(ii) is a weaker Lewis acid than Cu(ii) and, thus, it can detach protons from ligands upon coordination in a manner less than Cu(ii). The overall charge state is expected to be less negative when Zn(ii) is bound to Aβ, compared to Cu(ii). Thus, Zn(ii) may induce Aβ aggregation more efficiently than Cu(ii).^19,90^ Therefore, both metal binding and ligand deprotonation are associated with the degree of Aβ aggregation.

### Metal-induced variation in the secondary structure of Aβ

Distinct effects of Cu(ii) and Zn(ii) on the change in the secondary structure of Aβ were monitored using a model peptide. Brezesinski and coworkers designed a coiled coil peptide (*i.e.*, i,i+7) to mainly adopt the α-helical conformation, as shown in Fig. 3b.^89^ Two His residues capable of metal binding were included at the i and i+7 positions of the peptide, considering the metal-binding mode of Aβ (Fig. 2a and d). The His6 and His13 residues of Aβ responsible for metal binding were also placed at the same positions. Three Val residues rendered the coiled coil peptide prone to transformation into the β-sheet structure within hours to days. Upon treatment of Cu(ii), the structural transition from α-helix to β-sheet was observed in the CD spectra in a time-dependent manner, while Zn(ii) did not allow the peptide to form a β-sheet and maintain the α-helical conformation. Mechanistic details have not been reported, but their distinct binding modes may induce a different influence on the secondary structure of the peptide.

## Impact of metal ions on the aggregation and toxicity of Aβ

The roles of metal ions in the aggregation of Aβ still remain elusive. The influence of metal ions on Aβ aggregation is valid based on the timescales for the reactions of the metal exchange between Aβ peptides (microseconds to seconds *in vitro*) as well as their aggregation (minutes to hours *in vitro*).^70,90,99–102^ When metal ions bind to Aβ, the secondary structure of the peptide is altered, which can modify its aggregation forming structured or amorphous aggregates.^19,103^ Depending on the aggregation extent and morphology, the resultant Aβ aggregates can exhibit toxic events in cellular environments. Furthermore, Aβ bound to redox-active Cu(i/ii) catalytically produces ROS leading to oxidative stress and cell death.^78–80^

### Alteration in the aggregation pathways of Aβ by metal ions

In this review, we introduce some examples of *in vitro* studies that illustrate the variation of Aβ aggregation by metal ions under physiologically relevant conditions (*e.g.*, pH 7.4 at 37 °C). Notionally, the formation of Aβ fibrils occurs more rapidly with sub-equimolar concentrations of metal ions, compared to that under metal-free conditions (*vide supra*). Yuan and coworkers demonstrated this concept according to the results obtained by the thioflavin-T (ThT) fluorescence assay and TEM.^104^ ThT is a fluorescence dye that detects β-sheet-rich aggregates.^13^ The ThT fluorescence intensity of Aβ_40_ with a sub-equimolar concentration of Cu(ii) [Cu(ii) : Aβ = 0.25 : 1] increased at the early stage of its aggregation, relative to that of metal-free Aβ_40_. The resultant Aβ aggregates were observed to be fibrils, indicating that Cu(ii) accelerated Aβ aggregation producing β-sheet-rich fibrils. The studies employing Aβ_42_ also displayed fibrillary morphology upon incubation with sub-equimolar Cu(ii) [Cu(ii) : Aβ = 0.1 : 1].^105^ The counterexamples were reported, however. Hemmingsen's and Goto's groups determined that the generation of β-sheet-rich Aβ_40_ aggregates was retarded in the presence of sub-equimolar amounts of Cu(ii) [Cu(ii) : Aβ = 0.1–0.55 : 1] with the extension of the lag phase.^106,107^ In addition, the studies with atomic force microscopy (AFM) presented the resultant Aβ aggregates with amorphous characteristics.^107^ TEM studies with Aβ_42_ showed some moderate results with a mixture of filamentous and amorphous morphologies with sub-equimolar Cu(ii) [Cu(ii) : Aβ = 0.6 : 1].^108^ In the case of Zn(ii), microscopic events upon Aβ_40_ aggregation were characterized by analyzing the fluorescence-based aggregation kinetics.^109^ As a result, sub-stoichiometric concentrations of Zn(ii) were revealed to reduce the elongation rate of Aβ_40_ [Zn(ii) : Aβ = 0.025–0.125 : 1], but not completely inhibit the formation of amyloid fibrils.^109^ NMR studies further elucidated that the *N*-terminal region in Aβ_40_ could be transiently folded surrounding Zn(ii), forming a metastable Zn(ii)–Aβ complex, which could subsequently modify the ends of fibrils where elongation takes place and retard amyloid fibrillization.^109^

Upon increasing the concentration of metal ions up to equimolar and supra-equimolar amounts, the metal-mediated dimerization of Aβ could occur *via* bridging, but nonspecific metal binding to Aβ could form amorphous aggregates (*vide supra*). These phenomena were monitored by the ThT and turbidity assays, CD spectroscopy, and microscopies.^104,105,110–113^ Compared to the observations under metal-free conditions, the ThT fluorescence intensity in the overall macroscopic aggregation steps of both Aβ_40_ and Aβ_42_ was decreased in the presence of Cu(ii) or Zn(ii) with a metal-to-Aβ ratio not less than 1 [Cu(ii) : Aβ = 1–25 : 1; Zn(ii) : Aβ = 1–3 : 1]. On the other hand, the turbidity of Aβ samples increased, suggesting that metal ions could induce the generation of ThT-undetectable aggregates.^104^ CD spectroscopic studies further manifested that metal ions lowered the β-sheet contents of Aβ aggregates.^110,112^ The morphology of the resultant Aβ aggregates, confirmed by TEM and AFM, was unstructured.^104,105,110,112,113^ Exley and coworkers reported that Aβ_42_ incubated with equimolar Cu(ii) indicated fibrils that could be converted to be amorphous when supra-equimolar Cu(ii) [Cu(ii) : Aβ = 10 : 1] is treated with the peptide.^108^ Further research to elucidate the mechanisms of Aβ aggregation with a range of concentrations of Cu(ii) has been carried out based on a quantitative analysis of aggregation kinetics. Heegaard and coworkers proposed the aggregation models of Aβ_40_ in the presence of Cu(ii) [Cu(ii) : Aβ = 0.25–5 : 1] by spectroscopies, microelectrophoresis, mass spectrometry, and microscopies.^102^ Aβ was bound to Cu(ii) (timescale, milliseconds to seconds), forming a Cu(ii)–Aβ complex with 1 : 1 stoichiometry. This complex either aggregated (timescale, minutes) or remained soluble over a long time (timescale, hours to days) depending on the metal-to-peptide stoichiometry. Oligomers containing Cu(ii) can have multiple conformations in a dynamic equilibrium, resulting in diverse morphologies of Aβ aggregates at the endpoint of aggregation studies. Esbjörner and coworkers investigated the aggregation mechanisms of Aβ_42_ with Cu(i/ii) [Cu(i/ii) : Aβ = 0.5–2 : 1] by the global fitting of specific mathematical models to experimental data from the ThT assay with and without Aβ_42_ seeds.^114^ As a result, Cu(i) and Cu(ii) were revealed to inhibit the primary nucleation mildly and elongation of Aβ_42_, respectively, but TEM images still showed amyloid fibrils. It should be noted that the results were unpredictable based on the aforementioned roles of metal ions in changing Aβ aggregation and the inconsistency in previously reported observations could be due to the heterogeneous nature of Aβ samples and various experimental conditions, including the concentration and source of Aβ (*e.g.*, synthetic and recombinant);^41^ an apparent fluorescence quenching in the presence of Cu(ii) needs to be carefully interpreted due to the Cu(ii)-induced inner filter effect.^102,114,115^

More specifically, according to the toxicity of soluble and structured Aβ oligomers (*vide infra*), the effects of metal ions on the formation of oligomeric species should be further studied. The aforementioned fluorescence assay and microscopies (*e.g.*, ThT assay and TEM) have limitations in detecting relatively small oligomers over large Aβ aggregates such as fibrils.^19,26,116,117^ Other methods can be used for monitoring Aβ oligomers.^118^ For example, gel electrophoresis with Western blotting is commonly used to analyze Aβ species based on their molecular weight distribution.^116,119^ Low molecular weight oligomers (*e.g.*, less than 70 kDa) that may be produced at the early aggregation stage can be resolved by this method.^120^ The conformation of Aβ aggregates can be further differentiated in Western blotting or dot blotting employing different antibodies [*e.g.*, anti-Aβ antibody (6E10), anti-amyloid oligomer antibody (A11); anti-amyloid fibril antibody (OC)].^26,118,120–122^ Moreover, electrospray ionization–ion mobility–mass spectrometry (ESI–IM–MS) is a technique capable of characterizing heterogeneous oligomers.^118,123^ The mass and cross-sectional area of Aβ species, observed by ESI–IM–MS, provide information on the size and conformation of Aβ oligomers. The size distribution of Aβ aggregates, including low molecular weight oligomers, can also be obtained by dynamic light scattering experiments.^124^ The studies that demonstrate the formation of toxic Aβ oligomers upon treatment of metal ions complementarily using the aforementioned techniques are very limited. These detailed investigations can assist in advancing our understanding of metal-associated Aβ aggregation.

### Disruption of cellular events by metal-associated Aβ aggregates

**Fig. 4:**
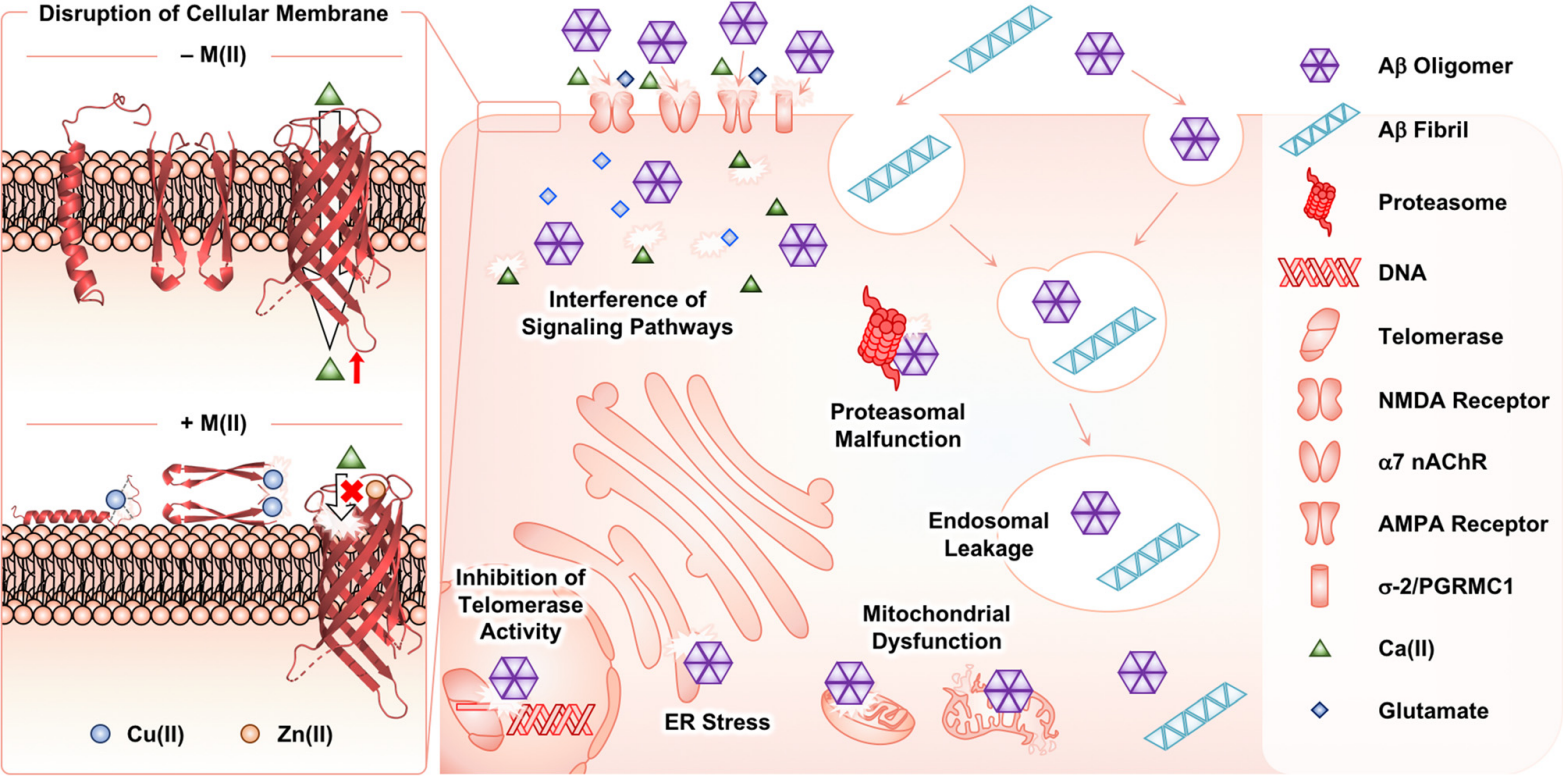
Possible toxic events induced by Aβ oligomers and fibrils with and without metal ions in cellular environments. In the absence of metal ions, Aβ species can interact with cellular membranes, changing their secondary structures at the surface or within the cell membranes. Consequently, Aβ can aggregate near the membrane and form a membrane-permeable pore structure that possibly controls the influx and efflux of neurotransmitters [*e.g.*, Ca(ii)]. Such processes can be altered upon interaction of Cu(ii) and Zn(ii) with Aβ species near the membranes. Extracellular Aβ aggregates can be internalized by endocytosis and the receptors in cellular membranes. The intracellular accumulation of Aβ aggregates can disrupt subcellular events.

Among Aβ aggregates, soluble and structured oligomers are the main species that trigger toxicity in cellular environments.^26^ Through secondary nucleation (Fig. 1f), Aβ fibrils can contribute to aggravating the cytotoxicity by proliferating the production of oligomers.^45–47^ The influence of metal ions on the Aβ-induced toxicity has been mainly proposed by investigating the interactions of Aβ with membranes in the absence and presence of metal ions. As illustrated in Fig. 4, Aβ species are known to contact cellular membranes, compromising their structural integrity.^26,125,126^ Under metal-free conditions, the interaction between Aβ peptides and membranes results in an equilibrium shift from the α-helical conformation to the β-sheet structure.^125,126^ This change in their secondary structure triggers the aggregation of Aβ near or within membranes, inducing the generation of membrane-penetrable pore structures, which can stimulate the leakage of Ca(ii) that is a neurotransmitter essential for signal transduction.^26,127,128^ Metal binding to Aβ near membranes is also suggested to govern the structural perturbation of membranes leading to toxicity. In the presence of negatively charged unilamellar vesicles (LUVs) as a model system mimicking cellular membranes, EPR studies indicated that Cu(ii) could bind to the *N*-terminal region of Aβ_42_.^129,130^ Upon Cu(ii) binding to Aβ in a solution of LUVs, its secondary structure was converted from β-sheet to α-helix, which possibly penetrates membrane bilayers, as monitored by CD spectroscopy.^129,130^ Dissimilar results were also reported depending on the type of artificial membranes. ^31^P and ^2^H solid-state NMR spectroscopic studies indicated that Cu(ii) without Aβ could destabilize the lipid layers of multilamellar vesicles and cause the formation of smaller vesicles.^131^ In the presence of Aβ_42_, this effect of Cu(ii) on the model membrane disappeared, possibly due to the competitive binding of Cu(ii) between lipid layers and Aβ. The studies using ^31^P solid-state NMR spectroscopy further revealed that Cu(ii)–Aβ_42_ could interact with the surface of negatively charged phospholipid membranes.^132^

Moreover, Cu(ii) can contribute to the dimerization of Aβ_42_*via* the formation of a His bridge.^129,133^ When Cu(ii) was treated with Aβ at a metal-to-peptide ratio greater than 0.6, EPR spectra exhibited characteristic *g* and *A* values, indicative of producing an Aβ dimer with a dinuclear Cu(ii) center. These spectral patterns were not observed for Aβ containing three His residues methylated at either the π- or τ-N atom of the imidazole side chain, highlighting the importance of His residues in the dimerization of Aβ. Solid-state NMR spectroscopic studies with LUVs that include ^31^P-labeled phospholipid head groups under experimental conditions generating the Cu(ii)-mediated His bridge presented the interaction between Cu(ii)–Aβ_42_ and the head groups of lipid membranes.^133^ This suggests the binding of Cu(ii)–Aβ_42_ at the surface of membranes rather than the insertion within the bilayers. Cu(ii)-bound His-bridged Aβ dimers are shown to be toxic in primary cortical neurons.^133^

When Aβ is further aggregated near membranes, and subsequently forms oligomers that can constitute a Ca(ii) channel inserted into membranes, Zn(ii) can interact with the channel. Rojas and coworkers illustrated that Zn(ii) could bind to Aβ_40_ oligomers incorporated into artificial membranes as a pore structure and inhibit the channel conductance, probably attenuating the toxicity induced by Aβ channels.^134^ Such behaviors of Zn(ii) toward Aβ oligomers were reversed by the addition of *o*-phenanthroline as a Zn(ii) chelator, corroborating the Zn(ii)-mediated blockade of Ca(ii) channels composed of Aβ oligomers. Similar results have been reported under various experimental conditions (*e.g.*, the type of membranes and pore structures composed of diverse lengths of Aβ).^135–140^ Upon further aggregation of Aβ oligomers into fibrils, the membranes were severely disrupted and, thus, the Ca(ii) selectivity of the Aβ channel was abolished. Zn(ii) did not stop such uncontrollable leakage of Ca(ii) from the channel.^141^

In addition to the surface of membranes, extracellular Aβ aggregates produced in the absence and presence of metal ions can interact with transmembrane proteins [*e.g.*, *N*-methyl-d-aspartate (NMDA) receptors, α7 nicotinic acetylcholine receptor (α7 nAChR), α-amino-3-hydroxy-5-methyl-4-isoxazolepropionic acid receptor (AMPA receptor), and σ-2 receptor and progesterone receptor membrane component 1 (σ-2/PGRMC1)], perturbing cellular signaling pathways.^142–146^ Upon internalization by the receptors and endocytosis, Aβ aggregates can lead to the dysfunction of cellular organelles and components, such as endosomes, mitochondria, endoplasmic reticulum (ER), proteasome, and telomerase.^147–151^

### Oxidative stress triggered by Cu(i/ii)–Aβ complexes

In the presence of a reducing agent, Cu(ii)–Aβ can participate in the generation of ROS (*vide supra*; Fig. 2e) and, consequently, impair cellular components through multiple pathways.^3,10–12,18,152,153^ In the brains of AD patients, an increased level of the products from the oxidation of lipids, proteins, and deoxyribonucleic acids (DNAs) is observed, compared to age-matched controls, which supports the relationship of oxidative damage to the pathology of AD.^154^ In particular, oxidative modifications of Aβ in a site-specific manner (*e.g.*, Asp, His, Phe, Tyr, and Met) are detected in the presence of Cu(ii) and reductants, which could modulate its aggregation and toxicity profiles.^78,155–157^ The Cu(ii)-triggered oxidation of Aβ_40_ is also available in membrane-mimicking environments, implicating the possibility of lipid peroxidation through ROS generated from Cu(i/ii)–Aβ near membranes with the consequent neuronal cell death.^158–160^ At the cellular level, the downstream response to ROS generation includes the impairment of protein expression, cell signaling, mitochondrial functions, and autophagy, as well as the promotion of inflammation and apoptosis.^154,161–164^

## Conclusions

A long journey in developing therapeutics targeting Aβ has only prompted the exploration of the intertwined pathology associated with Aβ and multiple pathological factors.^3^ On the basis of heterogeneous Aβ aggregates that are generated upon its self-assembly, a relationship between microscopic and macroscopic processes involved in Aβ aggregation has been recently investigated to establish the aggregation mechanisms.^34–39^ Increasing evidence suggests different facets of Aβ connected with metal ions: metal coordination to Aβ and metal-mediated aggregation and toxicity of Aβ.^3^ This highlights bioinorganic aspects of metal–Aβ complexes to illuminate the roles of metal ions in the Aβ-related pathology. Research endeavors discussed in this review can pave the way for elucidating the pathology of AD as well as developing effective therapeutics in the future.

## Conflicts of interest

There are no conflicts to declare.

## Supplementary Material
